# Data on influence of different nitrogen fertilizer rates and plant density on grain yield and yield components of Water Efficient Maize (WEMA) variety

**DOI:** 10.1016/j.dib.2020.105582

**Published:** 2020-04-18

**Authors:** Abidemi Ruth Adebayo, Erick Tshivetsi Sebetha

**Affiliations:** Faculty of Natural and Agricultural Sciences, Food Security and Safety Niche Area Research Group, North-West University Mafikeng Campus, Private Bag x 2046, Mmabatho 2735, South Africa

**Keywords:** WEMA maize, Grain yield, Plant density, Localities

## Abstract

Plant density and applications of nitrogen fertilizer have been recognized as the main crop management techniques to improve maize yield. The data showed effect of different nitrogen fertilizer rates and plant density on grain yield and yield components of water efficient maize. A Field experiment was conducted during the 2015/16 and 2016/17 planting seasons in two (Taung and Mafikeng) localities of North–West Province, South Africa to evaluate the influence of N fertilizer rates and plant density on grain yield and yield components of Water Efficient Maize (WEMA) variety. The experiment was laid out in split plot fitted into a randomized complete block design with four replicates in each site. The main plot effect was three plant densities (33333, 44444 and 55555 plants/ha) and nitrogen rates (0, 60, 120, 180 and 240 kg N ha^−1^) constituted the subplot. The parameters measured were grain yield and grain yield components. Data were analyzed with analysis of variance (ANOVA) of GenStat 11th edition. Differences in the treatment means were tested by Duncan Multiple Range Test (DMRT) at 5% level of probability. Regression and correlation analyses were used to determine relationship between grain yield, yield components and nitrogen rates.

Specifications table

**Table:** 

Subject	Agronomy and Crop Science
Specific subject area	Crop production, crop nutrition and soil fertility
Type of data	Figure Table
How data were acquired	Maize plants were harvested within the middle row from each plot with harvested area of 8 m^2^. The ears were air dried for six weeks after harvesting until uniform moisture content of 12% was attained. The ears were threshed manually.
Data format	Raw data
Parameters for data collection	Maize plants were harvested within the middle row from each plot with harvested area of 8 m^2^. The ears were air dried for six weeks after harvesting until uniform moisture content of 12% was attained. The ears were threshed manually. Data on yield and its components were evaluated as described in this article.
Description of data collection	The grain yield, stover yield, biological, shelling percentage, one thousand seed weight and grain ratio was obtained as described in this article.
Data source location	The experiment was carried out at the Molelwane, North-West University (NWU) Research Farm (25^0^ 48^1^S, 45^0^ 38^1^ E.; 1012 m asl) and Taung Experimental Station (27 30^1^S, 24 30^1^E; 1111 m asl) of the Provincial Department of Agriculture Both sites are located in the North West Province of South Africa.
Data accessibility	Raw data are attached as supplementary file.

## Value of the data

• The data showed the effect of different nitrogen fertilizer rates on grain yield and yield components.
• The data revealed the effect of plant density on grain yield and yield components.
• The data indicated the effect of interaction of nitrogen fertilizer rates, plant densities and locations on grain yield and yield components.
• The data can be used by crop nutritionist and general agronomist.

## 1 Data description

The data shows the influence of different nitrogen fertilizer rates and plant density on grain yield and yield components of Water Efficient Maize (WEMA) variety under different field conditions. The experiment was carried out during 2015/16 and 2016/17 planting seasons. The meteorological data of experimental locations (Table 1). The effect of each treatment factors on grain yield, total shoot biomass yield, stover, yield and harvest index were presented in Table 2. Table 3 indicates the influence of each treatment factors on shelling percentage, grain/cob ratio and one thousand seed weight. The interaction effect of location, plant densities and nitrogen fertilizer rates on grain yield, total shoot biomass, stover yield and harvest index as presented in Table 4. Table 5 displays the interaction effect of location, plant densities and nitrogen fertilizer rates on harvest index, grain/cob ratio and one thousand seed. Table 6 exhibits relationship between grain yield, yield components and nitrogen rates. Table 7 presents correlation relationship between grain yield and yield components. The supplementary data indicates the raw dataset of grain yield and yield components (Excel sheet 1). Also, supplementary data shows analyzed that obtained from raw data to estimate relationship between grain yield, yield components and nitrogen rates (Excel sheet 2–4).

**Table 1:** Effect of treatments factors on harvest index, shelling % 1000 seeds and grain/cob ratio of WEMA.

Treatment factors	Harvest index	Shelling %	Thousand seed mass	Grain/cob ratio
**Location**				
Molelwane	0.44b	78.2a	301.01b	2.45b
Taung	0.60a	70.0b	371.68a	4.30a
LSD _(0.05)_	0.02	0.6	0.46	0.02
**Plant density (Plants/ha)**				
33,333	0.53a	73.40b	340.64a	3.55b
44,444	0.50b	73.30a	332.34c	2.96c
55,555	0.54a	75.70a	336.05b	3.62a
LSD (p≤0.05)	0.02	0.70	0.57	0.03
**N rates (kg/ha)**				
0	0.54a	77.90a	328.31d	2.83e
60	0.51c	72.50c	332.70c	3.43c
120	0.47d	74.30b	332.28c	3.22d
180	0.55a	72.90c	342.18b	3.93a
240	0.53b	73.10c	346.28a	3.47b
LSD (p≤0.05)	0.02	1.00	0.73	0.04

Means with the same letter on the same column and treatment are not significantly different at P ≤ 0.05. using least different significant difference (LSD).

^⁎⁎^Significant at 5% probability.

**Table 2:** Interaction effect of N rates, plant density and location on grain yield and biomass yield.

	Grain yield t/ha						Biological yield t/ha					
	Molelwane Trial			Taung Trial			Molelwane Trial			Taung Trial		
*N* rates kg/ha	33,333	44,444	55,555	33,333	44,444	55,555	33,333	44,444	55,555	33,333	44,444	55,555
0	3.88	3.57	2.95	4.38	4.74	5.15	6.31	5.50	6.16	7.04	8.27	7.56
60	4.01	3.43	3.33	4.93	5.21	5.03	6.74	6.07	6.14	7.56	8.90	7.77
120	3.94	4.30	4.30	4.94	5.44	5.40	7.65	7.59	8.32	7.30	8.82	9.76
180	4.48	3.78	4.11	5.40	4.85	5.72	8.07	7.50	6.94	8.13	9.51	7.50
240	4.05	4.42	4.31	5.60	5.25	4.60	6.44	7.05	7.54	9.08	8.23	8.65
**LSD _(_*p*≤_0.05)_**	2.29						3.44

**Table 3:** Interaction effect of N rates, plant density and location on stover yield.

	Stover yield t/ha					
	Molelwane Trial			Taung Trial		
*N* rates kg/ha	33,333	44,444	55,555	33,333	44,444	55,555
0	2.43	2.55	2.59	2.66	3.13	2.82
60	2.72	2.74	2.70	2.64	3.86	2.55
120	3.72	3.31	4.03	2.37	3.44	4.32
180	3.60	3.38	3.16	2.70	3.80	2.74
240	2.38	2.74	3.12	3.50	3.68	3.41
**LSD (p≤0.05)**		1.64

**Table 4:** Interaction effect of N rates, plant density and location on harvest index and biomass yield.

	Harvest Index						Shelling %					
	Molelwane Trial			Taung Trial			Molelwane Trial			Taung Trial		
*N* rates kg/ha	33,333	44,444	55,555	33,333	44,444	55,555	33,333	44,444	55,555	33,333	44,444	55,555
0	0.52	0.45	0.46	0.62	0.59	0.62	84.00	72.00	94.00	70.00	70.00	77.00
60	0.22	0.57	0.48	0.53	0.57	0.70	82.00	79.00	78.00	61.00	67.00	68.00
120	0.45	0.25	0.37	0.68	0.48	0.57	76.00	73.00	79.00	73.00	73.00	71.00
180	0.49	0.45	0.44	0.67	0.60	0.66	68.00	85.00	76.00	72.00	69.00	69.00
240	0.51	0.50	0.46	0.60	0.54	0.60	77.00	76.00	95.00	71.00	69.00	70.00
**LSD(p≤_0.05)_**			0.02						44.56

**Table 5:** Interaction effect of *N* rates, plant density and location on 1000 seeds and grain/cob ratio.

	1000 Seeds (g)						Grain/cob ratio					
	Molelwane Trial			Taung Trial			Molelwane Trial			Taung Trial		
*N* rate kg/ha	33,333	44,444	55,555	33,333	44,444	55,555	33,333	44,444	55,555	33,333	44,444	55,555
0	307.13	295.36	277.91	377.00	352.75	359.75	2.20	3.20	0.18	3.90	4.70	2.81
60	304.42	288.62	281.68	377.25	370.25	374.00	2.22	2.75	1.76	4.15	4.96	4.78
120	298.04	298.72	291.07	369.00	373.63	363.25	2.27	2.58	2.25	3.80	3.96	4.44
180	301.13	322.43	312.80	376.00	378.25	362.25	5.57	1.93	2.21	4.36	5.58	3.94
240	313.69	309.98	312.24	382.75	370.50	388.50	2.36	2.64	2.65	4.71	3.88	4.60
LSD**(*p*≤_0.05)_**			3.5						0.21

**Table 6:** Relationship between grain yield, yield components and *N* fertilizer rates.

Parameters	Equations	*R*²
Grain yield	*y* = −0.0607*x*^2^ + 0.5233*x* + 3.612	0.95**
Biological yield	*y* = −0.1679*x*^2^ + 1.2881*x* + 5.582	0.85**
Stover yield	*y* = −0.1114*x*^2^ + 0.7846*x* + 1.958	0.75**
Harvest index	*y* = 0.01*x*^2^− 0.058*x* + 0.584	0.36^ns^
Shelling %	*y* = 0.5714*x*^2^−4.3486*x* + 80.9	0.67**
1000 seeds	*y* = 0.6957*x*^2^ + 0.3677*x* + 327.59	0.94**
Grain/cob ratio	*y* = −0.0857*x*^2^ + 0.6923*x* + 2.242	0.66**

**Table 7:** Correlation relationship between biomass yield and other parameters.

	*GY*	*BY*	*SY*	*HI*	*SH*	*TSW*	*GCR*
GY	1.00						
BY	0.93⁎⁎	1.00					
SY	0.07^ns^	0.43^ns^	1.00				
HI	0.66⁎⁎	0.50⁎⁎	-0.38^ns^	1.00			
SH	0.01^ns^	0.06^ns^	0.12^ns^	0.14^ns^	1.00		
TSW	0.80⁎⁎	0.74⁎⁎	0.06^ns^	0.61⁎⁎	-0.25	1.00	
GCR	0.74⁎⁎	0.67⁎⁎	0.00^ns^	0.62⁎⁎	-0.50⁎⁎	0.77⁎⁎	1.00

GY = Grain yield, BY = Biological yield, SY = Stover yield, HI =Harvest index, SH = Shelling %, TSW= Thousand seed weight, GCR = grain/cob ratio.

⁎⁎ ≤ 0.01, *≤ 0.05 and ns non- significant**.**

## 2 Experimental design, materials, and methods

### 2.1 Water efficient Maize for Africa

Water Efficient Maize for Africa (WEMA) is a drought-tolerant maize variety grown in Africa, particularly in the Southern African Development Community (SADC). It is purposely bred to cope with increasing drought conditions brought about by climatic variability in many parts of Africa. It was launched in 2008 by the African Agricultural Technology Foundation (AATF), and was developed through conventional breeding, but speeded up by marked assisted selection procedures. It is a partnership project between AATF, Monsanto's, and the National Agricultural Research Institute (NARS). Target countries for its use include Kenya, Mozambique, South Africa, Uganda and Tanzania. The major aim behind the development of this variety, as opposed to the common varieties, was to increase yields by 20–30% under moderate drought conditions and by 12–24% under high intensity drought conditions. The first three varieties were released in 2014. WE3127 variety was among the first three varieties released in South Africa [1].

### 2.2 Description of study area

**Fig. 1:**
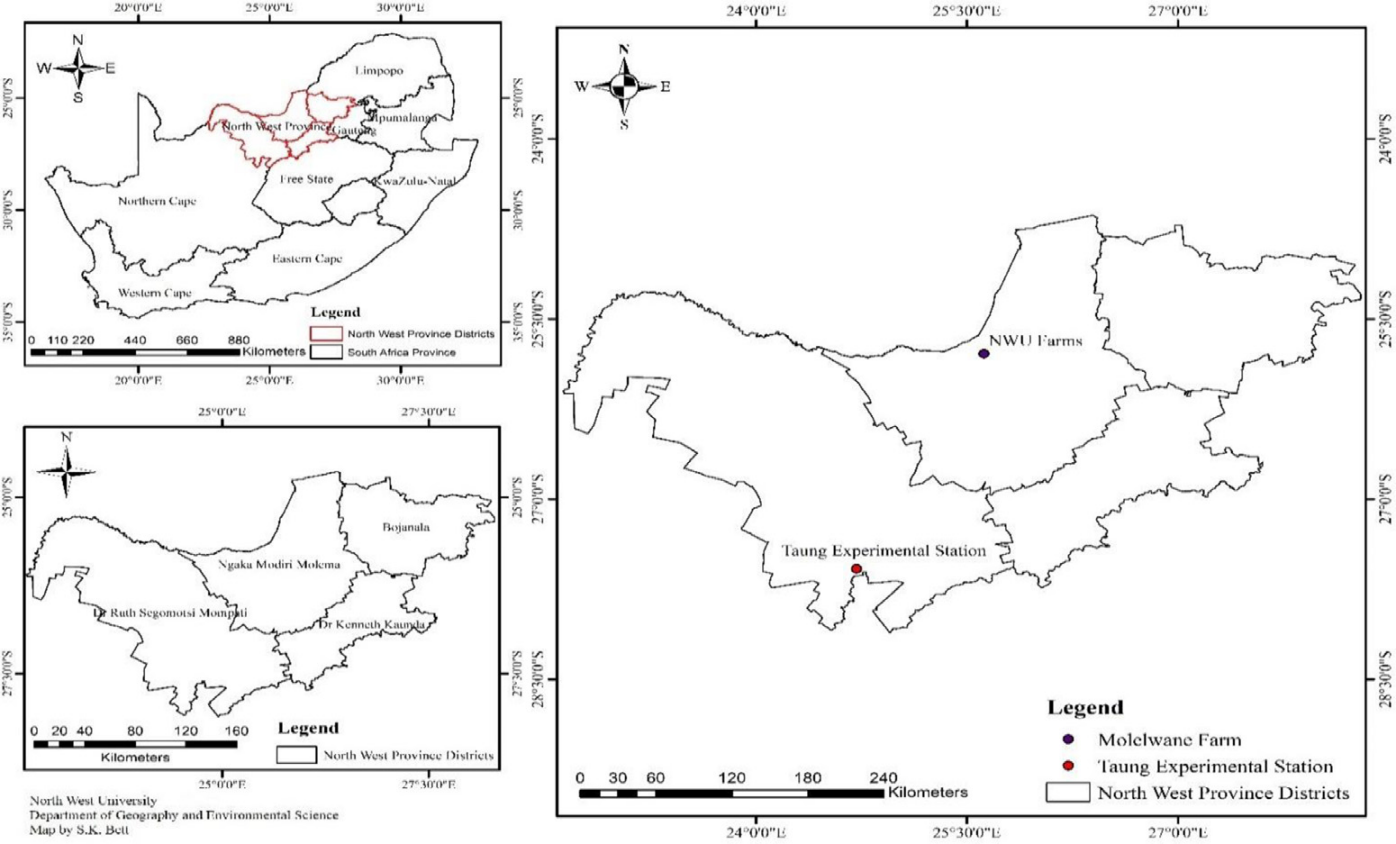
Map of North West Province, South Africa showing field trial sites.

The experiment was carried out at the Molelwane, North-West University (NWU) Research Farm (25^0^ 48^1^S, 45^0^ 38^1^ E.; 1,012 m asl) and Taung Experimental Station (27 30^1^S, 24 30^1^E; 1,111 m asl) of the Provincial Department of Agriculture Research Station during 2015/2016 and 2016/2017 planting seasons respectively. Both sites are located in the North West Province of South Africa (Fig. 1). The soil of North–West University (NWU) Research Farm belongs to Ferric Luvisol type and soil of Taung Experimental Station was classified as Rhodic Ferralsol type. The chemical properties of Ferric Luvisol are pH (4.41) total N (0.13%), available P (43 mg/kg) and K (241 mg/kg). However, the Rhodic Ferralsol had the following chemical properties, pH (5.38), total N (0.10 %), available P (27 mg/kg) and K (207.5 mg/kg) across two planting seasons.

**Fig. 2:**
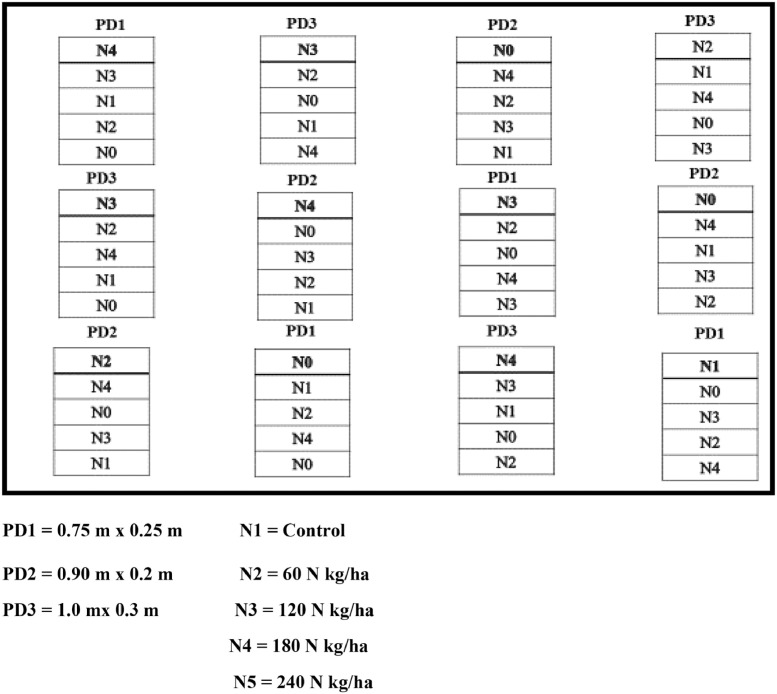
Field layout of plant density and nitrogen fertilizer rate.

The experimental sites were ploughed and harrowed two after ploughing. The layout of the experiment at each location was in the split plot arrangement fitted into a randomized complete block design with four replicates. The main plot effect was the three plant densities (33,333, 44,444 and 55,555 plants/ha) while the five N fertilizer rates (0.60, 120,180 and 240 kg N/ha) constituted the sub plot effect. Each subplot measured 6 m x 4 m with a total experimental plot size of 30 m × 76 m (0.228 ha) at each site (Fig. 2). The distance of 1 m and 2 m were maintained between plots and replicates respectively. Each experimental site has sixty subplots. Maize (WE 3127) seed sowing was done at inter and intra row spacing of 1m × 0.3m, 0.75 m × 0.3m and 0.9 m × 0.2 m to achieve the density of 33,333, 44, 4444 and 55,555, respectively by drilling method. The fertilized was applied in split method, by applying 30% of the each rate as basal treatment at planting using NPK 20:7:3 while 40% and 30% remaining quantity from each rate was applied as top dressing at 3 and 5 weeks after sowing (WAS) using lime ammonium nitrate (LAN, 28%). Both fertilizer types are granular type. Weeding was done manually at 3 and 7 weeks after sowing.

### 2.3 Data collection

Maize plants were harvested within the middle row from each plot with harvested area of 8 m^2^. The ears were air dried for six weeks after harvesting until uniform moisture content of 12% was attained. The ears were threshed manually. Data on yield and its components were evaluated as follows:

Yield $=\frac{Dry\;yield}{100-moisture\;content/100}$ (CIMMYT, 2013; [2])

Stover yield $=\frac{Stover\;yield\;obtained\;from\;harvested\;area}{harvested\;area}x\;actual\;plot\;area$

Total biological yield = grain yield + stover yield [3]

Harvest Index $=\frac{Economic\;yield(kg)}{Total\;bio\log icalyield(kg)}$ [4]

Shelling percentage $=\frac{Grain\;weight\;of\;shelledears}{Weight\;of\;unshelled\;ears}$ x 100

Grain /cob ratio = $\frac{Dry\;weight\;grain}{Dry\;weight\;of\;ears-dryweight\;grain}x100$ (CIMMYT, 2013)

While thousand seed weight was obtained with aid of weighting scale

### 2.4 Statistical analysis

All data obtained were subjected to analysis of variance (ANOVA) using the GenStat 11th edition. Differences between the treatment means were separated using Duncan Multiple Range Test (DMRT) test at 5% level of probability. Regression and correlation were used to estimate relationship between N rates, grain yield and yield components using Excel program.

## Conflict of Interest

Regarding the publication of this manuscript, the author declare no conflicts of interest.

## Appendix Supplementary materials

Image, application 1

Image, application 2

Image, application 3
