# Supplementary material for: Data on influence of different nitrogen fertilizer rates and plant density on grain yield and yield components of Water Efficient Maize (WEMA) variety
Source: Data Brief. 2020 Apr 18;30:105582. doi: 10.1016/j.dib.2020.105582 (PMC7200769; doi:10.1016/j.dib.2020.105582)
Supplement: Supplementary file 2 [file mmc2.docx]

**Table 1: The meteorological data of experimental locations**

|  | **2015/16 planting season** | | **2016/17 planting season** | | **2015/16 planting season** | | **2016/17 planting season** | |
| --- | --- | --- | --- | --- | --- | --- | --- | --- |
|  | **Molelwane Trial** | | | | **Taung Trial** | | | |
| **Months** | **Temperature (°C)** | **Rainfall**  **(mm)** | **Temperature (°C)** | **Rainfall**  **(mm)** | **Temperature (°C)** | **Rainfall**  **(mm)** | **Temperature (°C)** | **Rainfall**  **(mm)** |
| December | 27.70 | 31.20 | 25.10 | 117.20 | 28.6 | 9.00 | 27.10 | 145.6 |
| January | 26.30 | 62.80 | 23.10 | 147.80 | 27.6 | 85.00 | 23.80 | 241.60 |
| February | 27.10 | 18.60 | 22.30 | 282.80 | 27.6 | 15.20 | 23.60 | 155.40 |
| March | 23.60 | 79.40 | 21.60 | 21.00 | 24.1 | 37.60 | 22.60 | 13.00 |
| April | 21.00 | 37.80 | 19.10 | 77.60 | 20.4 | 61.80 | 18.30 | 42.60 |
| May | 15.90 | 17.20 | 15.60 | 0.00 | 15.7 | 22.60 | 15.20 | 0.60 |
| June | 13.60 | 10.40 | 14.30 | 0.00 | 13.3 | 0.00 | 12.40 | 0.00 |
| Total Mean | 22.17 | 36.77 | 20.16 | 92.34 | 22.47 | 33.03 | 20.42 | 85.54 |

Source: South African Weather Service (2018)

**Table 2: Effect of treatments factors on grain, biological, stover yield and harvest index of WEMA**

|  | **Yield ( kg/ha)** | | | Harvest  Index |
| --- | --- | --- | --- | --- |
|  | Grain | Total shoot biomass | Stover |  |
| **Location** |  |  |  |  |
| Molelwane | 3,092b | 6,930b | 3,010b | 0.44b |
| Taung | 5,110a | 8,270a | 3,170a | 0.60a |
| LSD (p≤0.05) | 60.00 | 90.00 | 70.00 | 0.02 |
| **Plant density (plants/ha)** |  |  |  |  |
| 33,333 | 4,500a | 7,430c | 2,870c | 0.53a |
| 44,444 | 4,480b | 7,740a | 3,260a | 0.50b |
| 55,555 | 4,560a | 7,630b | 3,140b | 0.54a |
| LSD (p≤0.05) | 70.00 | 110.00 | 80.00 | 0.02 |
| **N (kg/ha)** |  |  |  |  |
| 0 | 4,110c | 6,800d | 2,700d | 0.54a |
| 60 | 4,320b | 7,190c | 2,870c | 0.51c |
| 120 | 4,710a | 8,240a | 3,530a | 0.47d |
| 180 | 4,730a | 7,940b | 3,230b | 0.55a |
| 240 | 4,700a | 7,830b | 3,140b | 0.53b |
| LSD (p≤0.05) | 90 | 140 | 110 | 0.02 |

N=N rates, PD=Plant density, L= Location, S= Planting season. Notes: Means with the same letter on the same column and treatment are not significantly different at P ≤ 0.05. using least different significant difference (LSD). ** significant at 5% probability

**Table 3: Effect of treatment factors on shelling percentage, grain/cob ratio and thousand seed weight of WEMA**

| **Treatment factors** | **Shelling %** | **Grain/cob ratio** | **Thousand seed weight (g)** |
| --- | --- | --- | --- |
| **Location** |  |  |  |
| Molelwane | 78.2a | 2.45b | 301.01b |
| Taung | 70.0b | 4.30a | 371.68a |
| LSD _(0.05)_ | 0.6 | 0.02 | 0.46 |
| **Plant density (Plants/ha)** |  |  |  |
| 33,333 | 73.4b | 3.55b | 340.64a |
| 44,444 | 73.3a | 2.96c | 332.34c |
| 55,555 | 75.7a | 3.62a | 336.05b |
| LSD (p≤0.05) | 0.7 | 0.03 | 0.57 |
| **N rates (kg/ha)** |  |  |  |
| 0 | 77.9a | 2.83e | 328.31d |
| 60 | 72.5c | 3.43c | 332.70c |
| 120 | 74.3b | 3.22d | 332.28c |
| 180 | 72.9c | 3.93a | 342.18b |
| 240 | 73.1c | 3.47b | 346.28a |
| LSD (p≤0.05) | 1.00 | 0.04 | 0.73 |

N = N rates, PD = Plant density and L = Location Notes: Means with the same letter(s) in the same column and treatment are not significantly different at P ≤ 0.05. ** significant at 5% probability.

**Table 4: Interaction effect of treatment factors rates on grain, biological and stover yields (kg/ha) and harvest index of WEMA maize**

| **N rates**  **Kg/ha** | **Plant density**  **Plants/ha** | **Yield (kg/ha)** | | **Total shoot Biomass (kg/ha)** | | **Stover yield**  **(kg/ha)** | | **Harvest**  **Index** | |
| --- | --- | --- | --- | --- | --- | --- | --- | --- | --- |
|  |  | **Molelwane** | **Taung** | **Molelwane** | **Taung** | **Molelwane** | **Taung** | **Molelwane** | **Taung** |
| **0** | **33,333** | 3,878 | 4,380 | 6,306 | 7,036 | 2,428 | 2,656 | 0.52 | 0.62 |
|  | **44,444** | 2,945 | 5,147 | 5,490 | 8,270 | 2,553 | 3,128 | 0.45 | 0.59 |
|  | **55,555** | 3,568 | 4,739 | 6,161 | 7,559 | 2,593 | 2,820 | 0.47 | 0.62 |
|  |  |  |  |  |  |  |  |  |  |
|  | **33,333** | 4,013 | 4,925 | 6,736 | 7,562 | 2,723 | 2,637 | 0.23 | 0.52 |
| **60** | **44,444** | 3,328 | 5,031 | 6,067 | 8,893 | 2,743 | 3,861 | 0.56 | 0.57 |
|  | **55,555** | 3,434 | 5,214 | 6,137 | 7,765 | 2,703 | 2,551 | 0.48 | 0.69 |
|  |  |  |  |  |  |  |  |  |  |
| **120** | **33,333** | 3,938 | 4,934 | 7,648 | 7,304 | 3,719 | 2,370 | 0.45 | 0.68 |
|  | **44,444** | 4,287 | 5,385 | 7,593 | 8,823 | 3,307 | 3,438 | 0.25 | 0.48 |
|  | **55,555** | 4,287 | 5,442 | 8,319 | 9,760 | 4,032 | 4,318 | 0.37 | 0.57 |
|  |  |  |  |  |  |  |  |  |  |
| **180** | **33,333** | 4,480 | 5,429 | 8,069 | 8,129 | 3,591 | 2,700 | 0.49 | 0.67 |
|  | **44,444** | 4,111 | 5,724 | 7,487 | 9,511 | 3,376 | 3,787 | 0.45 | 0.54 |
|  | **55,555** | 3,775 | 4,848 | 6,937 | 7,493 | 3,162 | 2,739 | 0.44 | 0.66 |
|  |  |  |  |  |  |  |  |  |  |
| **240** | **33,333** | 4,054 | 5,585 | 6,437 | 9,080 | 2,383 | 3,495 | 0.51 | 0.60 |
|  | **44,444** | 4,306 | 4,559 | 7,050 | 8,234 | 2,744 | 3,675 | 0.50 | 0.54 |
|  | **55,555** | 4,417 | 4,480 | 7,536 | 8,653 | 3,119 | 3,407 | 0.46 | 0.60 |
| LSD (0.05) |  | 115.90 |  | 344.40 |  | 212.1 |  |  |  |

**Table 5: Interaction effect of plant density x nitrogen fertilizer rates x location on shelling percentage, grain: cob ration and 1000 seed weight**

| **N rates**  **Kg/ha** | **Plant density**  **Plants/ha** | **Shelling % (%)** | | **Grain: cob ratio** | | **1000 seed weight** | |
| --- | --- | --- | --- | --- | --- | --- | --- |
|  |  | **Molelwane** | **Taung** | **Molelwane** | **Taung** | **Molelwane** | **Taung** |
| **0** | **33,333** | 78.47 | 75.45 | 2.20 | 3.90 | 307.13 | 377.00 |
|  | **44,444** | 73.71 | 71.54 | 3.20 | 4.70 | 295.36 | 352.75 |
|  | **55,555** | 77.51 | 93.69 | 0.18 | 2.81 | 277.91 | 359.75 |
|  |  |  |  |  |  |  |  |
|  | **33,333** | 76.12 | 67.30 | 2.22 | 4.15 | 304 | 377.25 |
| **60** | **44,444** | 73.71 | 71.54 | 2.75 | 4.97 | 288.62 | 370.25 |
|  | **55,555** | 68.38 | 78.19 | 1.80 | 4.78 | 281.68 | 374.00 |
|  |  |  |  |  |  |  |  |
| **120** | **33,333** | 71.91 | 77.18 | 2.27 | 3.80 | 298.04 | 369.00 |
|  | **44,444** | 67.86 | 78.22 | 2.58 | 3.97 | 298.72 | 373.63 |
|  | **55,555** | 72.59 | 77.74 | 2.25 | 4.44 | 291.07 | 363.25 |
|  |  |  |  |  |  |  |  |
| **180** | **33,333** | 70.00 | 69.36 | 5.57 | 4.36 | 301.13 | 376.00 |
|  | **44,444** | 79.16 | 74.44 | 1.93 | 5.58 | 322.43 | 378.25 |
|  | **55,555** | 69.54 | 75.15 | 2.21 | 3.94 | 312.80 | 362.25 |
|  |  |  |  |  |  |  |  |
| **240** | **33,333** | 71.19 | 77.08 | 2.36 | 4.71 | 313.69 | 382.75 |
|  | **44,444** | 69.32 | 76.47 | 2.64 | 3.88 | 309.98 | 370.50 |
|  | **55,555** | 69.86 | 74.42 | 2.65 | 4.59 | 312.24 | 388.50 |
| **LSD (0.05)** |  | 1.48 |  | 0.10 |  | 3.52 |  |

**Table 6: Relationship between grain yield, yield components and N fertilizer rates**

| **Parameters** | **Equations** | **R²** |
| --- | --- | --- |
| Grain yield | y = -0.0607x^2^ + 0.5233x + 3.612 | 0.95** |
| Biological yield | y = -0.1679x^2^ + 1.2881x + 5.582 | 0.85** |
| Stover yield | y = -0.1114x^2^ + 0.7846x + 1.958 | 0.75** |
| Harvest index | y = 0.01x^2^ - 0.058x + 0.584 | 0.36^ns^ |
| Shelling % | y = 0.5714x^2^ - 4.3486x + 80.9 | 0.67** |
| 1000 seeds | y = 0.6957x^2^ + 0.3677x + 327.59 | 0.94** |
| Grain/cob ratio | y = -0.0857x^2^ + 0.6923x + 2.242 | 0.66** |

**Table 7: Correlation relationship between biomass yield and other parameters**

|  | *GY* | *TSB* | *SY* | *HI* | *SH* | *TSW* | *GCR* |
| --- | --- | --- | --- | --- | --- | --- | --- |
| GY | 1.00 |  |  |  |  |  |  |
| TSB | 0.93** | 1.00 |  |  |  |  |  |
| SY | 0.07^ns^ | 0.43^ns^ | 1.00 |  |  |  |  |
| HI | 0.66** | 0.50** | -0.38^ns^ | 1.00 |  |  |  |
| SH | 0.01^ns^ | 0.06^ns^ | 0.12^ns^ | 0.14^ns^ | 1.00 |  |  |
| TSW | 0.80** | 0.74** | 0.06^ns^ | 0.61** | -0.25 | 1.00 |  |
| GCR | 0.74** | 0.67** | 0.00^ns^ | 0.62** | -0.50** | 0.77** | 1.00 |

GY = Grain yield, TSB = Total shoot biomsss, SY = Stover yield, HI =Harvest index, SH = Shelling %, TSW= Thousand seed weight, GCR = grain/cob ratio. ** ≤ 0.01, * ≤ 0.05 and ns non- significant**.**
